# Expanded Somatic Mutation Spectrum of MED12 Gene in Uterine Leiomyomas of Saudi Arabian Women

**DOI:** 10.3389/fgene.2018.00552

**Published:** 2018-12-14

**Authors:** Ghada M. A. Ajabnoor, Nesma Amin Mohammed, Babajan Banaganapalli, Layla Saleh Abdullah, Ola Nabeel Bondagji, Nisma Mansouri, Nora Naif Sahly, Venkatesh Vaidyanathan, Nabeel Bondagji, Ramu Elango, Noor Ahmad Shaik

**Affiliations:** ^1^Department of Clinical Biochemistry, Faculty of Medicine, King Abdulaziz University, Jeddah, Saudi Arabia; ^2^Princess Al-Jawhara Al-Brahim Center of Excellence in Research of Hereditary Disorders, King Abdulaziz University, Jeddah, Saudi Arabia; ^3^Department of Genetic Medicine, Faculty of Medicine, King Abdulaziz University, Jeddah, Saudi Arabia; ^4^Department of Pathology, Faculty of Medicine, King Abdulaziz University, Jeddah, Saudi Arabia; ^5^Department of Gynecology and Obstetrics, Faculty of Medicine, King Abdulaziz University, Jeddah, Saudi Arabia

**Keywords:** uterine leiomyomas, MED12 gene, Arab women uterine, somatic mutations, Luteinizing hormone

## Abstract

MED12, a subunit of mediator complex genes is known to harbor genetic mutations, (mostly in exon 2), causal to the genesis of uterine leiomyomas among Caucasian, African American, and Asian women. However, the precise relationship between genetic mutations vs. protein or disease phenotype is not well-explained. Therefore, we sought to replicate the MED12 mutation frequency in leiomyomas of Saudi Arabian women, who represents ethnically and culturally distinct population. We performed molecular screening of MED12 gene (in 308 chromosomes belonging to 154 uterine biopsies), analyzed the genotype-disease phenotype correlations and determined the biophysical characteristics of mutated protein through diverse computational approaches. We discovered that >44% (34/77) leiomyomas of Arab women carry a spectrum of MED12 mutations (30 missense, 1 splice site, and 3 indels). In addition to known codon 44, we observed novel somatic mutations in codons 36, 38, and 55. Most genetically mutated tumors (27/30; 90%) demonstrated only one type of genetic change, highlighting that even single allele change in MED12 can have profound impact in transforming the normal uterine myometrium to leiomyomas. An interesting inverse correlation between tumor size and LH is observed when tumor is positive to MED12 mutation (*p* < 0.05). Our computational investigations suggest that amino acid substitution mutations in exon-2 region of MED12 might contribute to potential alterations in phenotype as well as the stability of MED12 protein. Our study, being the first one from Arab world, confirms the previous findings that somatic MED12 mutations are critical to development and progression of uterine leiomyomas irrespective of the ethnic background. We recommend that mutation screening, particularly codon 44 of MED12 can assist in molecular diagnostics of uterine leiomyomas in majority of the patients.

## Introduction

Uterine leiomyomata (UL), commonly referred as leiomyomas or fibroids arises from smooth muscle cell lining of the uterus, called myometrium (Khan et al., 2014). They occur in approximately up to 30% of women above 30 years and also affect about 70% of women by the age of 50 years (Stewart, 2001; Zimmermann et al., 2012; Eltoukhi et al., 2014). Based on their myometrial origin, leiomyomas are categorized into sub-serosal, intramural, or sub-mucosal types. The number of leiomyomas per uterus can be single or multiple (leiomyomatosis) (Parker, 2007; Gupta et al., 2008; Metwally et al., 2011). These tumors are monoclonal and contain a large amount of extracellular matrix (ECM, proteoglycan, fibronectin) that is surrounded by a thin pseudo-capsule of areolar tissue and compressed muscle fibers (Moore et al., 2010). The size of UL varies, ranging from microscopic to very large (over 10 cm) (Parker, 2007; Gupta et al., 2008). Although benign in nature, UL causes a great deal of morbidity including excessive uterine and vaginal bleeding, acute pelvic pain, and pressure on adjacent organs and severe pregnancy complications (Baird et al., 2003; Medikare et al., 2011; Chang et al., 2013; Moroni et al., 2015). UL are the common cause for one third of gynecology hospital admissions and hysterectomy, thus causing considerable burden on the health care system (Ligon and Morton, 2000; Wallach and Vlahos, 2004; Parker, 2007; Medikare et al., 2011; Cardozo et al., 2012; Shen et al., 2015).

Specific molecular mechanisms underlying the genesis of leiomyomas have remained relatively unknown but there have been some suggestions regarding contributory role of age, race, early menarche, obesity, nulliparity, and steroid hormones in leiomyoma predisposition (Parazzini, 2006; Terry et al., 2010; Tropeano et al., 2012; Brohl et al., 2015; Stewart et al., 2017; Vercellini and Frattaruolo, 2017; Pavone et al., 2018). Cytogenetic studies have shown that 40 to 50% of leiomyomas harbor chromosomal abnormalities (Islam et al., 2013; Maltese et al., 2017). In addition to this, multiple studies have reported different types of mutations, altered expression and/or methylation status of hormone receptor genes, growth factors, repair genes, extracellular matrix genes, collagen and mitochondrial genes in uterine leiomyomas (Islam et al., 2013; Montgomery et al., 2014; Sant'anna et al., 2017). However, their mixed findings led to confusion in ascertaining the causal relation of genetic factors contributing to leiomyoma development (Leppert et al., 2006; Parker, 2007; Dimitrova et al., 2009; Moore et al., 2010; Islam et al., 2013; Montgomery et al., 2014; Laganà et al., 2017). With the advent of whole exome sequencing, somatic mutations in mediator complex subunit 12 gene (MED12) have been first reported in leiomyomas from Finnish Caucasian patients (Mäkinen et al., 2013). Subsequent studies on African or Mixed ancestry South African patients yielded similar findings (Mäkinen et al., 2011a,b). Variety of MED12 mutations including missense, in-frame insertion-deletions, and intrinsic type variations were found in different leiomyoma phenotypes (Mäkinen et al., 2011a,b, 2014a; Halder et al., 2014; Osinovskaya et al., 2016; Sadeghi et al., 2016; Heinonen et al., 2017; Wang et al., 2017). It is of particular interest, to note that all reported mutations were mostly localized to exon 2, underlining its potential contribution in the genesis of uterine leiomyomas (Barbieri et al., 2012; Mäkinen et al., 2013; Schwetye et al., 2014; Osinovskaya et al., 2016; Lee et al., 2018).

Interethnic variations in the prevalence of uterine leiomyomas, as well as its tumor characteristics (nodes number and size), have been noticed in African-American women (Parker, 2007; Okolo, 2008). This suggests the need to have ethnic or population specific genetic data to gain insight into the disease etiology. In Saudi Arabia and other Middle East Arab countries, prevalence and incidence of leiomyomas was initially undermined by gynecologists. But recent reports suggests that UL accounts for significant proportion of all gynecological morbidities seen in women of Arabian ethnicity (Rouzi et al., 2001; Sayyah-Melli et al., 2009; Ibrar et al., 2010; Sait et al., 2014; Yerushalmi et al., 2014; Ulrich, 2015). Given the high consanguinity rate and rapid changes in diet and life styles, MED12 sequencing of leiomyomas from Arab women is expected to present unique genetic alterations. Owing to the lack of studies, present study has aimed to establish the role of somatic mutation in MED12 gene in leiomyomas of Saudi patients. Furthermore, to ascertain if somatic mutations elicit phenotypic changes at protein level, this study has built the 3-dimension protein structure and analyzed the structural differences between native and mutant MED 12 models.

## Methods and Materials

### Clinical Sampling

This study was approved by Institutional Ethics Committee on Human Research of King Abdulaziz University Hospital (KAUH) before its commencement. A total of 154 fresh tissue specimens (comprising of leiomyoma and matched myometrium tissues) belonging to 77 hysterectomized uterine samples were collected from department of Histopathology at KAUH, Jeddah. Tissue biopsies from uterine hysterectomies were collected and examined by the consultant pathologist to confirm the histopathological presentation of fibroids. Tissue (1–10 mg approximately) collection was done in small vials filled with normal saline and stored at −70°C until they were processed for genetic analysis. The basic anthropometric details (height, weight, BMI), clinical details (symptoms, co-morbidities, age at hysterectomy), and biochemical profile (Prolactin, Luteinizing hormones, Estradiol, Progesterone, Total Cholesterol, Tumor Size, and BMI) of patients were collected from hospital data repository, which maintains individual case histories for at least one decade. Patient's informed consent was obtained prior to their sample inclusion in to this investigation. Individual case records revealed that all patients were Saudi by ethnic origin and prior to their hysterectomy all of them have undergone series of clinical and radiological examinations to conclude the evidences of fibroids.

### MED12 Genetic Screening

#### DNA Extraction and PCR

DNA was extracted from all tissue samples by using the QIAamp DNA Mini Kit (Qiagen, Hilden, Germany) according to the manufacturer's instructions. Total DNA concentration and purity was quantified using the NanoDrop (Thermo Scientific NanoDrop™ ND-2000 1-position Spectrophotometer USA) at an absorbance of 260 nm. Extracted DNA was tested for its integrity using 0.8% agarose gel electrophoresis. The wild type human gene sequences of MED12 exon 2 were retrieved from online databases of *Ensembl Genome Browser* (http://www.ensembl.org/Homo_sapiens) and *National Center for Biotechnology Information* (http://www.ncbi.nlm.nih.gov/gene). The primer sets (Forward 5′-AACGTAAGGGCCCAGCTTTA-3′; Reverse 5′-CAGGGCCTTTGCTCCTTCTTA-3′) flanking the exons 2 region of MED12 gene were designed *in-house* using *Primer3 software* (http://frodo.wi.mit.edu/). The 40 cycle PCR for MED12 amplification was carried out using 1 μl of human genomic DNA (40 ng) in a 50 μl reaction mixture which contained 1 unit of Taq DNA polymerase, 20 to 30 pmol of forward and reverse primers, 1 mM of magnesium chloride and 2.5 of 10X ammonium sulfate buffer. Thermal cycle conditions for amplification PCR consisted of 1st step-3 min cycle of initial denaturation at a temperature of 93°C, followed by 2nd step- consisting 45 cycles each of 40 s of denaturation at 93°C, annealing at 57–62°C and primers extension at 72°C, and 3rd step: final extension or polymerization at 72°C for 10 min. After the PCR reaction, all products were electrophoresed on 2% agarose gel, followed by its analysis in an UVitec Gel Documentation system-232 (UVitec, Cambridge, UK) for imaging the gel and to determine the amplicon lengths.

#### Sanger Sequencing, Sequence Alignment, and Mutation Identification

For targeted DNA sequencing, PCR products of amplified exon were purified using QIAquick PCR Purification Kit (cat. Nos. 28104) following its manufacturer instructions. The purified PCR products were used as DNA template for cycle sequencing reaction with *ABI Prism Big-Dye Terminator Cycle Sequencing Ready Reaction Kit v1.1* (Life Technologies, USA). The sequencing PCR was carried out by using 1 μl of template DNA (20 ng) in a 10 μl reaction mixture which contained 1 μl ready reaction mix (Life Technologies, USA), 1 μl reaction buffer (Life Technologies, USA), 20 pmol of either forward or reverse primer. Then all the PCR products were analyzed by capillary electrophoresis in the ABI-Prism 3730xl Genetic Analyzer (Applied Biosystems, USA). BioEdit (version 6.0.7) and mixed sequence reader sequence alignment programs were used for aligning wild-type and patient's sequences in order to annotate sequence mismatches. Somatic mutations were those sequence changes which were specific to leiomyomas but not adjacent normal myometrium tissues.

### Computational Pathogenicity Predictions

#### Missense Mutations

We screened all the missense mutations using different computational algorithms like Scale-Invariant Feature Transform (SIFT), Polymorphism Phenotyping-2 (PolyPhen-2), Combined Annotation Dependent Depletion (CADD), and Functional Analysis through Hidden Markov Models (FATHMM) to measure their deleterious potential. Basic operational information about these computational methods is described below.

#### SIFT

This is a sequence-homology based web server (SIFT; http://sift.jcvi.org/) which accepts mutation query in the form of both chromosome and allelic changes to predict potentially deleterious mutations (tolerance index score of ≤ 0.05 SIFT prediction scores range from 0 to 1). The deleteriousness of mutation refers to the potential phenotypic and functional changes it could cause in corresponding protein molecules.

#### PolyPhen-2.0

This is a probabilistic classifier which calculates functional significance of an allele change by Naive Bayes, a set of supervised learning algorithms (http://genetics.bwh.harvard.edu/pph2/) to identify the deleterious mutations. The input options for this method constitute the database aceession number or protein sequence along with variant details. All the mutations with ≥0.9 PSIC scores (Position-specific independent count) are classified as “possibly damaging mutations.”

#### CADD

CADD v1.2 is pre-computed score database (http://cadd.gs.washington.edu) which predicts the pathogenic variants using integrative annotation methods. This method accepts variant call format (VCF) of mutations as an input to generate the combined annotation score (c-score) as an output. The top 10% functional variants demonstrate a c-score of ≥10, and top 1% damaging variants shows the C-score of ≥20 and most pathogenic top 0.1% variants demonstrate the c-score of ≥C30.

#### FATHMM

FATHMM (http://fathmm.biocompute.org.uk/) is computational method which predicts the functional effects of both intronic and exonic variants. It accepts the input details like chromosome number, position, reference and alternate bases, and provides outputs in the form of *p*-values (range from 0 to 1). The deleterious mutations will possess the prediction score of ≥0.5 and benign or neutral mutations will have the prediction score of ≤ 0.5.

### Structural and Stability Analysis of MED12 Mutations

In order to better understand the functional impact of missense mutations at 3 dimensional level, we have simulated the protein structure through *ab-initio* method [using I-Tasser and Modeller9v3 web servers], owing to absence of crystal structure or identical templates of MED12 protein structure in publicly available biological databases (Banaganapalli et al., 2016). The 3-D dimensional models for both wild type and mutant protein models were constructed by manual insertion of specific amino acid changes in the primary protein sequence of MED12. The structural divergence in MED12 protein due to amino acid substitutions was compared by initial superimposition of Cα traces and backbone atoms of wild and mutant protein forms, followed by calculation of the RMSD value differences among equivalent atoms using Pymol. Structural deviance of molecules was calculated in terms of RMSD scores, which is < 0.2 for amino acids and < 2.0 for proteins. We analyzed the stability of both native and mutant forms of MED12 protein through mCSM method (Banaganapalli et al., 2016).

### Statistical Analysis

Statistical analysis was performed with statistical package of social sciences (SPSS) (version 21.0) software for Windows. Categorical variables of disease characteristics are presented in the form of percentages, whereas continuous variable data was presented as mean and standard (mean ± SD) deviations. The differential correlation between individual tumor category and tumor relevant clinical, anthropometric, and biochemical characteristics were compared by Pearson Correlation Coefficient method. For all tests, significance level was set to *p* < 0.05 at 95% confidence limit.

## Results

### Genetic Analysis

**Figure 1:**
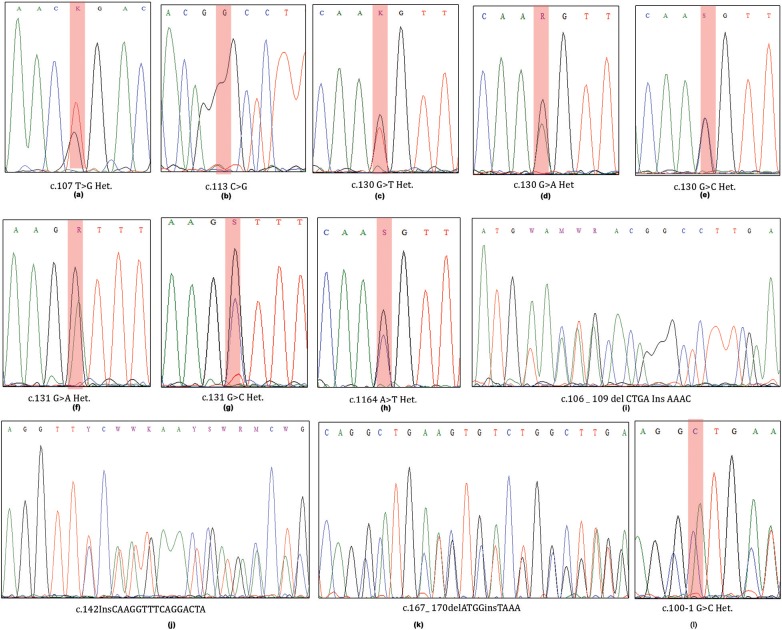
ABI Sequenced chromatograms showing exon2-MED12 somatic mutations at codons 36, 44, and 55 (**a–h**; missense mutations) and at codons 36, 47, and 56 (**i–l**; heterozygous Indels).

A total of 34 (44.15%) out of 77 cases harbored 12 different types of somatic mutations in exon 2 region of MED12 gene (Table 1; Figure 1). Of these cases, 30 (38.96%) individuals have demonstrated missense mutations, 1 (1.29%) case with splice site acceptor mutation and the remaining 3 (3.89%) showed insertion-deletion mutations. Single and double missense mutations were noticed in 27/30 (90%) cases and 3/30 cases (10%), respectively.

**Table 1:** The list of Exon 2-MED12 somatic mutations identified in leiomyomas of Saudi women.

**No**	**Positive leiomyoma cases**	**Nucleotide change**	**Variant type**	**Genetic code**	**Amino acid change**
1	1	c.107 T>G.	Missense	CTG>CGG	p.Leu36Arg
2	1	c.113C>G	Missense	GCC>GGC	p.Ala38Gly
3	5	c.130 G>A.	Missense	GGT>AGT	p.Gly44Ser
4	3	c.130 G>C.	Missense	GGT>CGT	p.Gly44Arg
5	2	c.130 G>T.	Missense	GGT>TGT	p.Gly44Cys
6	13	c.131 G>A.	Missense	GGT>GAT	p.Gly44Asp
7	1	c.131 G>C.	Missense	GGT>GCT	p.Gly44Ala
8	4	c.164 A>T.	Missense	GAG >GTG	p.Glu55Val
9	1	c.100-1 G>C	Splice Acceptor	—	
10	1	c.167_ 170delATGGinsTAAA	Indel	—	p. H56 _G57del Ins p.L56_N57
11	1	c.142InsCAAGGTTTCAGGACTA	Frame shift	—	p.Q47fs (Q48fs)
12	1	c.106 _ 109 del CTGA Ins AAAC	Indel	—	p.L36_37T del p.K36_P37

#### Single Nucleotide Mutations

We have observed 8 different types of heterozygous somatic missense mutations in leiomyomas. Interestingly, up to 80% (24/30) of the base changes we observed in Leiomyomas were specific to codon 44 (130 and 131 nucleotides) of MED12 coding transcript (ENS-100000374080). Of the codon 44 mutations, c.131G>A (13/24; 54.16%) is predominantly prevalent over c.130G>A (5/24; 20.83%), c.130G>C (3/24; 12.5%), c.130G>T (2/24; 8.33%), and c.131G>C (1/24; 4.16%) base substitutions. Additional missense mutations (novel ones) were located at codon 36 [c.107 T>G (1/30; 1.29%)], codon 38 [c. 113 C>G (1/30; 1.29%)], and codon 55 [c.164 A>T (4/30; 5.19%)]. One novel splice site loss mutation c.100-1 G>C (1/30; 1.29%) was seen to result in exon-2 skipping in the coding transcript.

#### Multiple Nucleotide Mutations

We observed 3 different insertion-deletions mutations in 3 UL cases (3/77; 3.89%). Of which, two indels (C.167_ 170delATGGinsTAAA & c.106_109delCTGAInsAAAC) were noticed in two separate cases (2/77; 2.59%), in addition to one case (1/77; 1.29%) with a frame shift mutation (c.142InsCAAGGTTTCAGGACTA). All these mutations were heterozygous and somatic in nature.

### Pathogenicity Prediction of Somatic Mutations by Computational Tests

The computational functional prediction analysis attributed pathogenicity to all missense mutations, supporting their important role in leiomyomagenesis (Table 2). All the missense mutations (8/8; 100%) were extremely intolerant with a SIFT score of 0.00 to 0.05 suggesting them to be damaging. Polyphen-2 analysis has also confirmed pathogenicity of these mutations (8/8; 100%), as their scores lied in the range of >0.9 to 1. CADD v1.2 analysis identifies damaging mutations based on their combined annotation scores (c-score for pathogenic mutations should be >25). CADD classified all mutations (8/8; 100%) as lethal owing to their high c-score (≥ 25) values. Confirming the above findings, FATHAMM analysis has also supported the damaging ability of MED12 missense mutations on its protein function (the prediction scores for all 8 mutations is in deleterious range ≥ 0.5 to 1).

**Table 2:** Pathogenicity prediction of MED12 Exon 2 somatic mutations by various *in-silico* algorithms.

	**Allele**	**CDS_position**	**Amino_acids**	**Codons**	**SIFT^a^**	**PolyPhen^b^**	**CADD_score^c^**	**FATHMM_score^d^**
X:71119380-71119380	T/G	107	p.Leu36Arg	cTg/cGg	0	0.997	26.8	0.77474
X:71119386-71119386	C/G	113	p.Ala38Gly	gCc/gGc	0.02	0.689	24.8	0.59056
X:71119403-71119403	G/A	130	p.Gly44Ser	Ggt/Agt	0	1	27.3	0.86901
X:71119403-71119403	G/C	130	p.Gly44Arg	Ggt/Cgt	0	1	26.4	0.87129
X:71119403-71119403	G/T	130	Gly44Cys	Ggt/Tgt	0	1	27	0.87129
X:71119404-71119404	G/A	131	p.Gly44Asp	gGt/gAt	0	1	26.9	0.87052
X:71119404-71119404	G/C	131	p.Gly44Ala	gGt/gCt	0	0.999	25.1	0.86901
X:71119437-71119439	A/T	164	p.Glu55Val	gAg/gTg	0	0.941	27.3	0.62785

a *SIFT ≤ 0.05 = Deleterious*.

b *PolyPhen >0.5–1 = Pathogenic*.

c *CADD_score ≥ 25 = Lethal*.

d *Fatham ≥ 0.5 = Damaging*.

#### MED12 Protein Structure 3D Modeling

**Figure 2:**
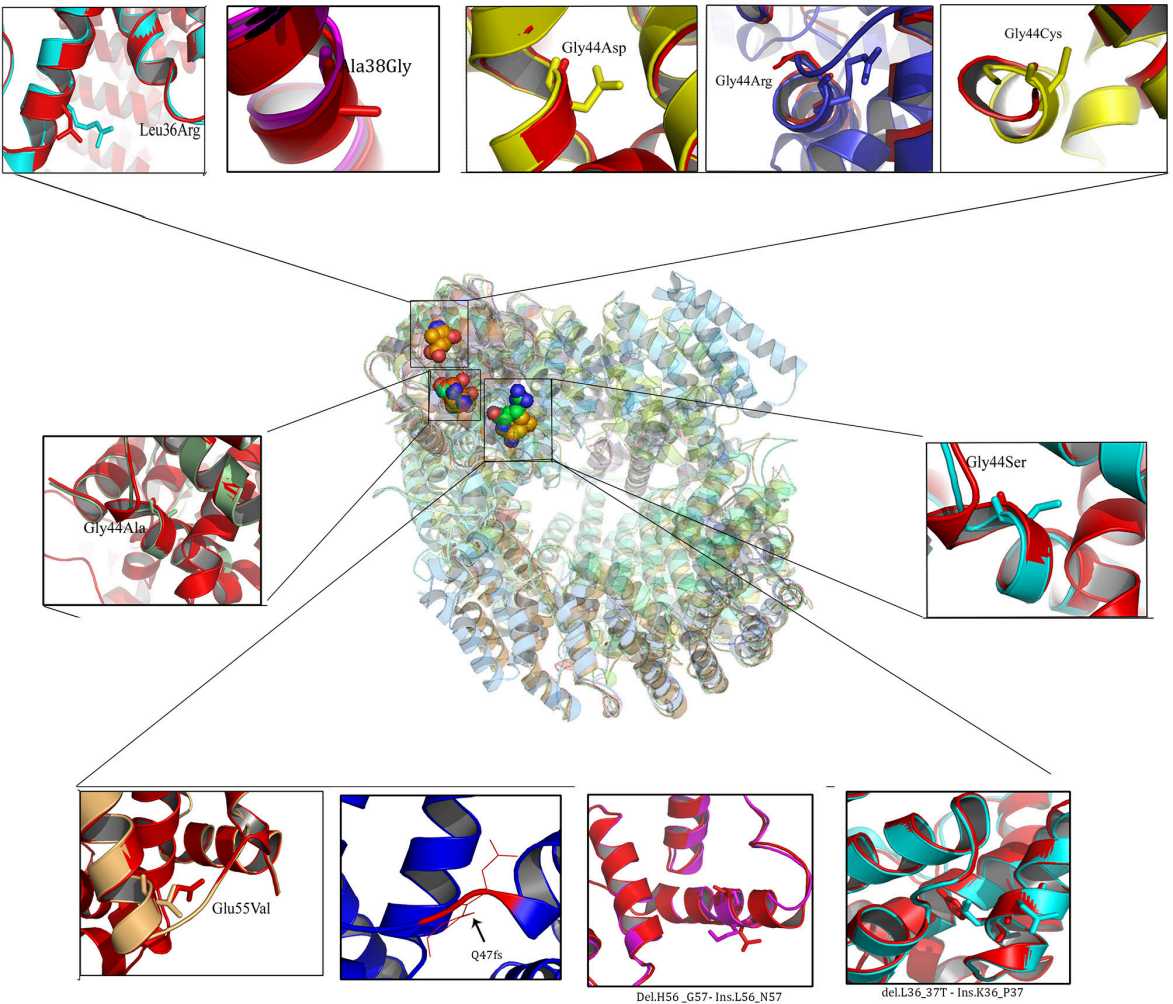
Superposed view of 3D models of native and mutant (Missense and Indels) MED12 proteins.

Owing to the limitations of I-Tasser web server in building 3- Dimensional protein structures of more than 1,500 amino acids, MED12 protein chain was initially modeled in two separate chains (1,000 and 1,021 aa) and later joined together using edit conf command in Gromacs tool (Figure 2). Both polypeptide chains possessed a confidence scores in −5 to +2 range, template modeling (TM) score of > +0.5 with the mean root mean square deviation (RMSD) score of 4.1 ± 3.0. Protein stereochemical quality testing (PROCHECK) showed that amino acids in disallowed region of MED12 protein are compliant to Ramachandran plot rule. The percentage of amino acid residues in core (allowed) and non-core (disallowed) regions of native MED12 protein are found to be 98.2 to 1.8%, respectively.

#### Protein Structural Divergence Analysis

The RMSD values of the c-alpha atoms of mutant against their wildtype amino acid residues (L36R, G38A, G44A, G44S, G44D, G44R, G44C, and E55V) revealed significant structural drift at residue level (0.32–0.58 Å) but not at polypeptide chain level (1.22–2.25 Å). Higher deviation (high RMSD number) value suggests the loss of hydrogen and ionic contraction between the carbon atom and functional groups in amino acid residues. This suggests that all eight MED12 amino acid substitutions are of deleterious nature and may likely to disrupt structural orientation of native protein. Interestingly, protein structures with amino acid substitutions at L36R, G44C, and G38A positions showed less structural divergence (0.32–0.40 Å) compared to the G44A, G44S, G44D, G44R, and E55V substitutions, which showed higher structural divergence (>0.50 Å). It is of particular interest to note that amino acid substitution corresponding to 44th (G → R; 58 and 2.25 Å) residue showed higher level of divergence compared to other mutations. Overall, the structural analysis results predicted that all ten MED12 mutations disrupt the orientation of native protein (Table 3).

**Table 3:** Structural deviations of native and mutant MED12.

**Amino acid variation**	**RMSD**	
	**Whole structural level**	**Amino acid level Å**
L36R	0.32	1.52
G38A	0.4	1.68
G44A	0.51	1.67
G44S	0.52	1.96
G44D	0.522	1.83
G44R	0.58	2.25
G44C	0.32	1.23
E55V	0.52	1.99
p. H56 _G57del Ins p.L56_N57	0.33	1.98
p.L36_37T del p.K36_P37	0.332	1.89

*RMSD : Structural deviance of molecules was calculated in terms of RMSD scores, which is < 0.2 Å for amino acids and < 2.0 Å for proteins*.

### Basic Clinical Characteristics of Uterine Leiomyoma Patients

**Figure 3:**
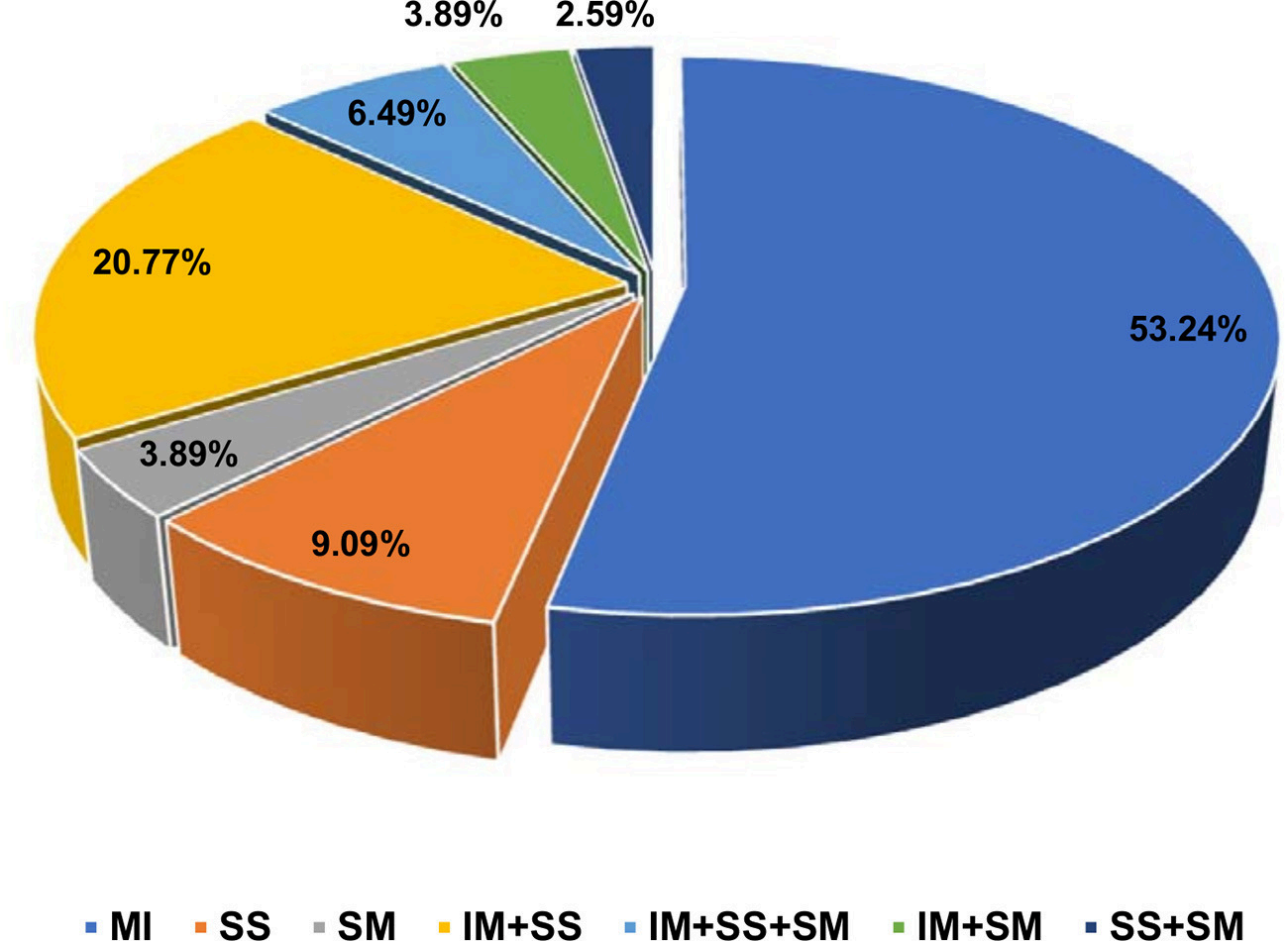
Pie chart of distribution of different histopathological types of leiomyomas. IM, intramural; SS, subserosal; SM, submucosal.

Overweight (BMI value is 25–29 kg/m^2^) and obesity (BMI value is >30 kg/m^2^) are the two most common (36 and 46%, respectively) anthropometric characteristics noticed among Arab uterine leiomyoma patients (Supplementary Table 1). Women in perimenopause (41–50 years; 53.2%) are the major age group who have undergone uterine hysterectomy due to leiomyomas compared to women in menopause (>51 years; 29.8% patients) or pre-menopause (29–40 years; 17% patients) stages. Majority of the patients showed single leiomyoma type (61/77; 79%), i.e., either intramural (41/77; 53.2%), submucosal (7/77; 9.09%), or subserosal (3/77; 3.89%). Remaining patients had mixed category tumors, i.e., tumor in more than one location (Figure 3). Majority of the women (62/77; 80.51%) had multiple leiomyoma nodes (>2 nodes).

### Association of MED12 Mutation Status With Clinical Characteristics of Leiomyoma Patients

**Figure 4:**
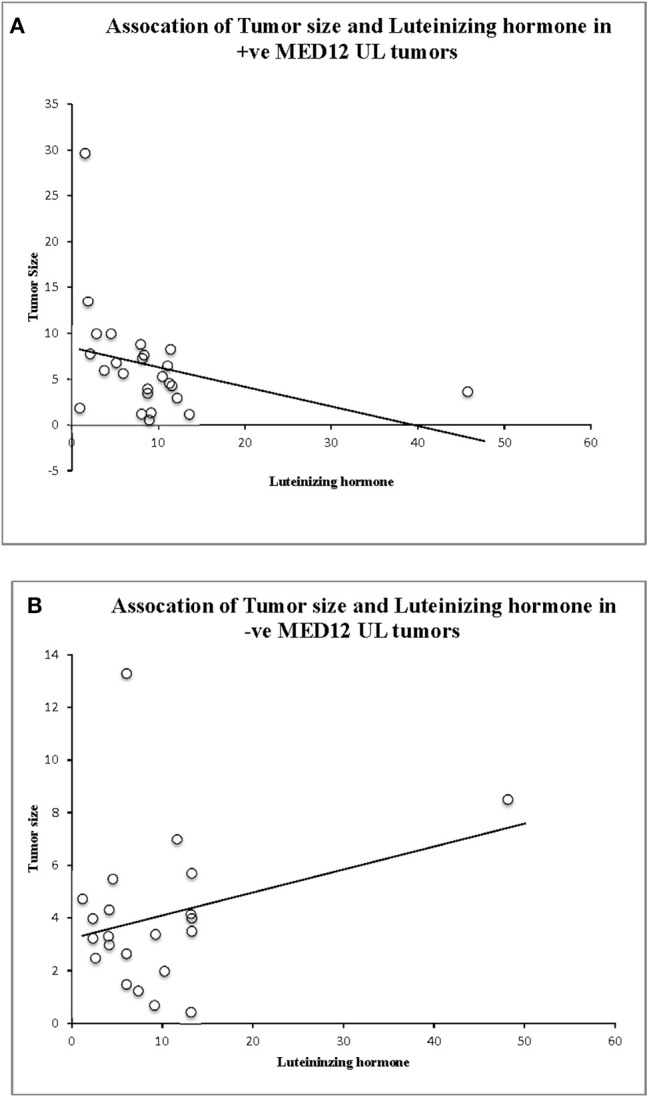
Association analysis of luteinizing hormone (LH) levels, tumor size, and MED12 genotype status. **(A)** Significant association between tumor size, LH and MED12 mutation positive tumors. **(B)** Lack of association between tumor size, LH, and MED12 mutation negative tumors.

We compared the biochemical characteristics like prolactin, luteinizing hormone (LH), estradiol, progesterone, and total cholesterol in leiomyoma patients to examine the influence of MED12 mutations on clinical and biochemical characteristics. Patients with MED12 mutation in leiomyoma have larger tumors (7.99 ± 3.9 cm; *p* = 0.02), high BMI (33.84 ± 6.9 kg/m^2^; *p* = 0.04) and low serum levels of luteinizing hormone (13.8 ± 8.4 mU/L; *p* = 0.02) compared to women who are not carrying the mutation (Table 4). Serum biochemical characteristics including prolactin, estradiol, progesterone and total cholesterol levels were comparable between women with and without MED12 mutations. Regarding the association analysis, a significant positive correlation in groups (+ve and −ve for MED12 mutations) between leiomyoma size with BMI, and prolactin (*p* for all tests is < 0.05; Table 4) were observed. However, luteinizing hormone showed significant inverse correlation with tumor size (*p* = 0.008) only in the MED12+ve group compared to the MED12 −ve group (Table 5; Figures 4A,B) This finding indicates that luteinizing hormone may have protective effect against tumor growth.

**Table 4:** Biochemical characteristics of UL patients (*n* = 77).

**Variable**	**UL −ve MED12 mean ± SD (*n* = 43)**	**UL +ve MED12 mean ± SD (*n* = 34)**	***P*-value**
Prolactin (ng/mL)	162.4 ± 121.4	202.6 ± 163.7	0.22
Luteinizing hormone (mU/L)	16.4 ± 8.4	12.09 ± 7.4	^*^0.021
Estradiol (Pmol/L)	180.3 ± 155.5	185.7 ± 147.35	0.85
Progesterone (ng/mL)	17.3 ± 7.6	17.6 ± 8.8	0.84
Total cholesterol (mmol/L)	5.3 ± 0.91	5.3 ± 1.23	0.92
Tumor size (cms)	4.7 ± 3.8	7.99 ± 7.9	^*^0.027
BMI (kg/m^2^)	30.88 ± 6.4	33.84 ± 6.9	^*^0.05

*Data is expressed as mean ± SD*.

* *Indicates statistically significant difference*.

**Table 5:** The correlation between tumor size (MED12 positive and negative) and different clinical characteristics of leiomyoma patients.

**Clinical variable**	**Tumor size**		**Tumor size**	
	**MED12 mutation positive women (*****n*** = **34)**		**MED12 mutation negative women (*****n*** = **43)**	
	**Pearson correlation (*r*)**	***P-*value**	**Pearson correlation (*r*)**	***P-*value**
BMI	0.53	^*^0.045	0.237	^*^0.033
Estradiol	0.145	0.38	0.041	0.791
LH	0.96	^*^0.008	−0.074	0.512
Total cholesterol	0.25	0.12	0.164	0.145
Progesterone	0.151	0.36	0.041	0.716
Prolactin	0.43	^*^0.007	0.404	^*^0.001

* *Correlation significance was considered at p < 0.05*.

## Discussion

**Figure 5:**
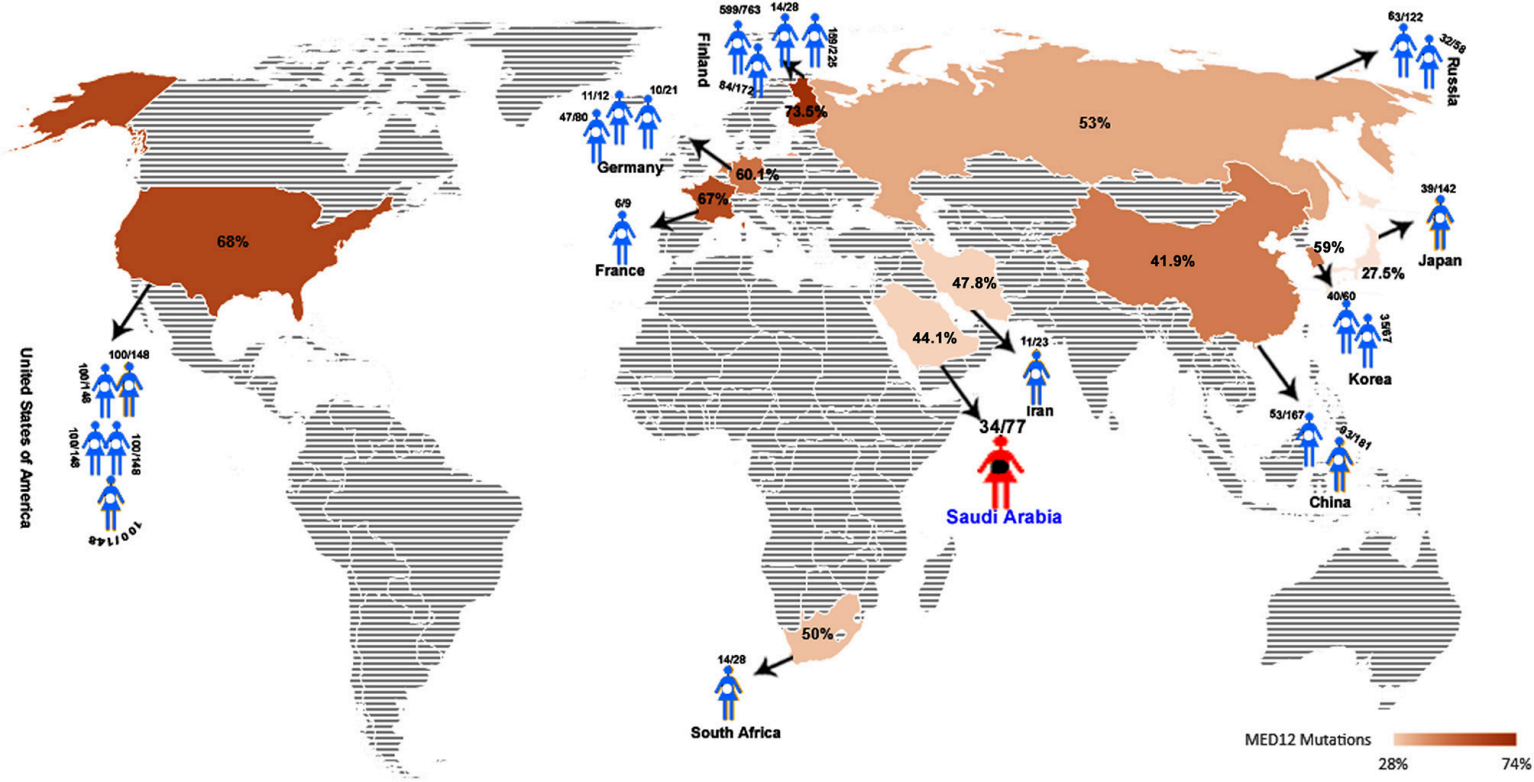
Global map showing the average UL-MED12 mutation frequency (in %) reported for different countries.

MED12 gene, mapped to Xq13.1, is known to show germline mutations all across its 45 exons in different clinical phenotypes like, neurodevelopmental and behavioral disorders (Banaganapalli et al., 2016). However, In leiomyomas, somatic mutations (be it missense, frameshifts, or Indels) in MED12, particularly in exon-2 region, are proposed to be one of the underlying cause of tumorigenesis (McGuire et al., 2012; Ravegnini et al., 2013; Halder et al., 2014; Kämpjärvi et al., 2016a; Sadeghi et al., 2016). So far, 20 independent investigations from different continents like North America (USA), Europe (Finland, Germany, Netherlands, France), Africa (South Africa), and Asia (China, Japan, and Korea) have identified MED12 somatic mutations in almost 1,616 uterine leiomyomas (out of 2,417 tumors tested; 66.85%). Figure 5 shows that the frequency of MED12 mutation is variable across different studies ranging from 27% (Matsubara et al., 2013) to as high as 92% (Rieker et al., 2013) (Supplementary information is provided in Supplementary Table 2).

Our study, being the first one from Middle Eastern Arab countries, confirms the trend of finding MED12 mutations in uterine leiomyomas. We observed that >80% of missense mutations were localized to codon 44 (89.28%), a rate higher to the reports of (Je et al., 2012) (66.6%) and (Mäkinen et al., 2011a,b) (49%) and less to the findings of Markowski et al. (2012) (95.8%) but similar to those of McGuire et al. study (89.5%) (Mäkinen et al., 2011a, 2014a,b; McGuire et al., 2012). We identified a codon 36 missense mutation in one patient, which, was previously reported (Rieker et al., 2013; Sadeghi et al., 2016; Maltese et al., 2017). Our additional interesting observation is that 16% of our missense mutation positive leiomyoma cases had a novel mutation spectrum (at codons 38 and 55). Noticeably, in 3/30 (10%) mutation positive cases, nucleotide changes were large indels, which further underscores the frequent genetic instability of MED12 gene in leiomyomas (Bertsch et al., 2014). Previous whole exome studies have revealed the heterozygous nature of somatic mutations in MED12 gene (Rieker et al., 2013; Bertsch et al., 2014; Di Tommaso et al., 2014). This came in agreement with this study, as most of the missense and indels mutations we observed were also in heterozygous state, in consistence with the fact that MED12 is a X-chromosomal gene, which is subjected to lyonization. Only one MED12 allele is expressed in each tumor cell due to random X-chromosome inactivation. This suggests us that even single allele change in MED12 gene can have profound impact in transforming the normal uterine myometrium to leiomyomas.

The ascertainment of causal genetic variants in disease pathogenesis is possible when strong genotype-disease phenotype correlations are observed. In this direction, previous reports indicate a positive correlation between MED12 mutation positivity with a larger tumor size in leiomyomas of South African origin (Mäkinen et al., 2011a, 2017). At the same time, leiomyomas of Finnish origin tended to be smaller in size (median 4 cm) than their South African counterparts (Mehine et al., 2013). MED12 mutated leiomyomas (with ≥5.5 cm in diameter) collected from South African women revealed the high number of mutations in large tumors compared to smaller ones (Mehine et al., 2013). Our results showed MED12 positive tumors are larger compared to those MED12 negative tumors. This suggests that MED12 mutations are the actual tumor triggering events in uterus.

The MED12 mutated leiomyomas show common and cellular type histopathological features over rare mitotically active, atypical and necrotic types (Mittal et al., 2015; Kämpjärvi et al., 2016b). This is consistent with our findings that MED12 mutations are seen in 84% of common and 16% of cellular leiomyomas. Nevertheless, some histopathological characteristics of leiomyosarcomas such as mitotically active cells have also appeared in leiomyoma cases with MED12 mutations, indicating possible transformation of leiomyoma toward malignancy when somatic mutations are accumulated. MED12 mutations are reported not just in benign uterine neoplasms but also in highly aggressive leiomyosarcomas (Markowski et al., 2012; Mäkinen et al., 2014b; Sadeghi et al., 2016; Yoon et al., 2017; Lee et al., 2018). Moreover elevated number of mitoses in leiomyomas may also result from hormonal factors, because these tumors frequently occur in pregnancy, during the secretory phase of the menstrual cycle, and in patients undergoing hormonal therapy (Walker, 2002; Wise et al., 2004; Salama et al., 2006). Luteinizing hormone (LH) is a glycoprotein hormone produced by human gonadotropin cells in the anterior pituitary gland. This hormone interacts with extracellular membrane portion of LH/hCG receptors and activates the signal transduction process, which is necessary for the hormonal functioning (Sarais et al., 2017). Few studies have highlighted the positive association between LH and leiomyoma development, wherein high LH levels are shown to contribute to the occurrence of large sized leiomyomas (≥4 cm) in premenopausal women (Horiuchi et al., 2000). Our findings show that high levels of LH are significantly correlated with large sized tumors carrying mutation. This suggests that the size of MED12 positive tumors is probably mediated through LH/hCG receptor proteins. Liao et al. (2012) have shown that higher LH in ovarian epithelial tumor cell lines upregulated the expression of VEGF (Vascular endothelial growth factor) and SLIT2 (Slit Guidance Ligand 2). VEGF and SLIT2 genes play an important role in ovarian cancer angiogenesis. In the present study, LH level is negatively correlated with tumor size when MED12 mutation is present. This supports the view that MED12 mutation is causal to UL. This indicates that the role of LH in UL development is minimal, if any, with reference to MED12 gene mutation.

Currently, one of the major challenges in interpreting genetic data is the classification of somatic mutations into drivers and passengers. In context of MED12, clustering of mutations at a distinct, evolutionarily conserved region establishes it to be a candidate driver gene. Moreover, MED12 mutation pattern also suggests it to be putative oncogene (Perot et al., 2012). Like other oncogenes, majority of MED12 mutations are located recurrently at the same amino acid position (codon 44), suggesting that glycine at codon 44 is mutation hotspot for leiomyomas and its location in evolutionarily conserved nucleotide sequences makes it a putative causal variant (Matam et al., 2015; Wang et al., 2015, 2017). Since, exon2 region falls in Cyclin C-CDK8 binding domain of MED12 protein, it is expected that mutations in this region induce conformational changes in MED12 binding interface of Cyclin C-CDK8 domain, which further negatively influence binding characteristics and stability of CDK8-mediator complex (Banaganapalli et al., 2016).

This study admits few limitations, including the smaller sample size, however, due to lack of any molecular data on leiomyomas from Saudi Arabian women, present study acts as an initiator for future large-scale population studies. We could not determine the age of onset of leiomyomas in our patient group because majority of leiomyomas are asymptomatic and gain clinical attention only when they grow large and start to manifest severe clinical complications. Based on our genetic findings, we recommend that screening exon-2 region mutations in leiomyomas can assist the molecular diagnostics of uterine tumors. For mutation negative leiomyomas, performing molecular tests for genes like HMGA1, steroid hormone receptors (Govindan et al., 2009; Shaik et al., 2009; Bondagji et al., 2017), fumarate hydratase (Vaidya et al., 2012), mitochondrial genes (Shaik et al., 2011a,b), etc., may give insights into the MED12-independent tumorigenesis mechanisms of ULs. The ascertainment of MED12 causal role in leiomyoma genesis can be done through functional biology assays or through gene knockout in animal models. The precise diagnosis of uterine leiomyomas can be made when MED12 genetic findings are coupled with clinical, biochemical, and histopathological findings. The frequent genetic alterations of MED12 gene in uterine leiomyomas, also raises the need to screen for its involvement in other gynecological neoplasms like endometriosis, ovarian tumors, and leiomyosarcomas etc.,

This study concludes that MED12 somatic mutations (44%) are by and large frequently observed in uterine leiomyomas of Saudi women. Of all mutations observed, codon-44 (seen in 89% tumors) seems to be a mutational hotspot region with plausible involvement in leiomyomagenesis. Furthermore, we observed that MED12 mutation status was correlated with tumor size and serum Luteinizing hormone levels. Our computational studies have predicted that MED12 mutations may affect its structural plasticity of the protein. In compliance to the previous findings about MED12 genetic alterations in leiomyomas of different ethnic groups, our study affirms that MED12 mutation positivity is critical to development and progression of ULs irrespective of ethnic background. Both clinical and molecular findings of our study can collectively add important information about the molecular etiological basis of leiomyomas, especially in the Arab world.

## Author Contributions

GA, RE, NB, NS, BB, LA, and NM: conceptualization; GA, NM, NS, BB, RE, and OB: data curation; GA and NAM: formal Analysis; RE: funding acquisition; GA, RE, NB, NS, and BB: Investigation; GA, NAM, NS, BB, and RE: methodology; NM, GA, and VV: project administration; GA, NAM, NS, BB, RE, and OB Resources; BB: software; GA, NS, NB, and RE: supervision; GA, NAM, NS, BB, RE, and OB: validation; BB: visualization; GA, NAM, NS, BB, and RE: writing original draft; GA, NAM, BB, LA, OB, NM, NA-S, RE, VV, NB, and NS: writing review and editing.

### Conflict of Interest Statement

The authors declare that the research was conducted in the absence of any commercial or financial relationships that could be construed as a potential conflict of interest.
