# Supplementary material for: Expanded Somatic Mutation Spectrum of MED12 Gene in Uterine Leiomyomas of Saudi Arabian Women
Source: Front Genet. 2018 Dec 14;9:552. doi: 10.3389/fgene.2018.00552 (PMC6302612; doi:10.3389/fgene.2018.00552)
Supplement: Supplementary file 1 [file Table_1.DOCX]

| Patient characteristics | UL | Mean ±SD |
| --- | --- | --- |
| Age | No. % |  |
| Premenopausal from 29 to 40 years | 13 (16.88 %) | 37.55 ±3.4 |
| Menopausal from 41 to 50 years | 41 (53.24 %) | 46.70 ±2.47 |
| Postmenopausal (> 51 years)  Total age groups from 29 to > 51 years | 23 (29.87 %)  77 (100%) | 55.5 ±2.46  47.6 ±6.9 |
| BMI | | |
| Normal weight from18.5 to 24.9 kg/m^2^ | 13 (16.88%) | 21.75 ±2.38 |
| Overweight From 25 to 29.9 kg/m^2^ | 28 (36.36%) | 27.43 ±1.33 |
| Obesity >30 kg/m^2^ | 36 (46.75%) | 37.6 ±5.92 |
| Total BMI groups from18.5 to >30 kg/m^2^ | 77 (100%) | 32.03 ±7.17 |

**Suppl Table 1: Age and BMI for UL patients. Age and BMI are expressed by percentage and Mean ±SD.**

| **References** | **Ethnicity** | **Frequency of MED12-exon 2 mutations** | |
| --- | --- | --- | --- |
|  |  | Mutation Positive Tumors /Total Tumors | **%** |
| (Lee *et al.*, 2018) | South Korean | 40/60 | 66.67% |
| (Heinonen *et al.*, 2017) | French | 599/763 | 79% |
| (Osinovskaya *et al.*, 2016) | Russian | 63/122 | 51.6% |
| (Sadeghi *et al.*, 2015) | Iran | 11/23 | 47.8 % |
| (Halder *et al.*, 2015) | Southern US | 92/143 | 64.3 % |
| (Schwetye *et al.*, 2014) | American | 15/28 | 54% |
| (Bertsch *et al.*, 2014) | American | 133/178 | 75% |
| (Heinonen *et al.*, 2014) | Caucasian/Finnish | 138/164 | 84% |
| (Ravegnini *et al.*, 2013) | American | 9/13 | 69% |
| (Markowski *et al.*, 2013) | German | 10/21 | 48% |
| (Matsubara *et al.*, 2013) | Japanese | 39/142 | 27.5% |
| (Makinen *et al.*, 2013) | Caucasian/Finnish | 84/172 | 48.8% |
| (de Graaff *et al.*, 2013) | Netherlands | 11/19 | 58% |
| (Rieker *et al.*, 2013) | German | 11/12 | 92% |
| (Je *et al.*, 2012) | Korean | 35/67 | 52% |
| (Perot *et al.*, 2012) | French | 6/9 | 67% |
| (McGuire *et al.*, 2012) | American | 100/148 | 68% |
| (Markowski *et al.*, 2013) | German | 47/80 | 59% |
| (Makinen, Mehine*, et al.*, 2011) | Finnish | 159/225 | 71% |
| (Makinen, Heinonen*, et al.*, 2011) | South African | 14/28 | 50% |
| (Dzhemlikhanova *et al.*, 2017) | Russia | 32/58 | 55.2%) |
| (Lien *et al.*, 2016) | China | 93/181 | 50.28% |
| (Ye *et al.*, 2015) | China | 53/167 | 31.7% |

**Suppl Table 2. Summary of studies presenting MED12 exon 2 mutation frequencies in leiomyoma patients of different ethnicities**

Bertsch, E., Qiang, W., Zhang, Q., Espona-Fiedler, M., Druschitz, S., Liu, Y., Mittal, K., Kong, B., Kurita, T. & Wei, J. J. (2014). *Modern pathology : an official journal of the United States and Canadian Academy of Pathology, Inc* **27**, 1144-1153.

de Graaff, M. A., Cleton-Jansen, A. M., Szuhai, K. & Bovee, J. V. (2013). *Hum Pathol* **44**, 1597-1604.

Dzhemlikhanova, L. K., Efimova, O. A., Osinovskaya, N. S., Parfenyev, S. E., Niauri, D. A., Sultanov, I. Y., Malysheva, O. V., Pendina, A. A., Shved, N. Y., Ivashchenko, T. E., Yarmolinskaya, M. I., Kakhiani, M. I., Gorovaya, E. A., Tkachenko, A. N. & Baranov, V. S. (2017). *J Clin Pathol* **70**, 233-236.

Halder, S. K., Laknaur, A., Miller, J., Layman, L. C., Diamond, M. & Al-Hendy, A. (2015). *Molecular genetics and genomics : MGG* **290**, 505-511.

Heinonen, H. R., Pasanen, A., Heikinheimo, O., Tanskanen, T., Palin, K., Tolvanen, J., Vahteristo, P., Sjoberg, J., Pitkanen, E., Butzow, R., Makinen, N. & Aaltonen, L. A. (2017). *Sci Rep* **7**, 1015.

Heinonen, H. R., Sarvilinna, N. S., Sjoberg, J., Kampjarvi, K., Pitkanen, E., Vahteristo, P., Makinen, N. & Aaltonen, L. A. (2014). *Fertil Steril* **102**, 1137-1142.

Je, E. M., Kim, M. R., Min, K. O., Yoo, N. J. & Lee, S. H. (2012). *Int J Cancer* **131**, E1044-1047.

Lee, M., Cheon, K., Chae, B., Hwang, H., Kim, H. K., Chung, Y. J., Song, J. Y., Cho, H. H., Kim, J. H. & Kim, M. R. (2018). *Int J Med Sci* **15**, 124-128.

Lien, H. C., Huang, C. S., Yang, Y. W. & Jeng, Y. M. (2016). *Histopathology* **68**, 433-441.

Makinen, N., Heinonen, H. R., Moore, S., Tomlinson, I. P., van der Spuy, Z. M. & Aaltonen, L. A. (2011). *Oncotarget* **2**, 966-969.

Makinen, N., Mehine, M., Tolvanen, J., Kaasinen, E., Li, Y., Lehtonen, H. J., Gentile, M., Yan, J., Enge, M., Taipale, M., Aavikko, M., Katainen, R., Virolainen, E., Bohling, T., Koski, T. A., Launonen, V., Sjoberg, J., Taipale, J., Vahteristo, P. & Aaltonen, L. A. (2011). *Science* **334**, 252-255.

Makinen, N., Vahteristo, P., Kampjarvi, K., Arola, J., Butzow, R. & Aaltonen, L. A. (2013). *Eur J Hum Genet* **21**, 1300-1303.

Markowski, D. N., Huhle, S., Nimzyk, R., Stenman, G., Loning, T. & Bullerdiek, J. (2013). *Genes Chromosomes Cancer* **52**, 297-304.

Matsubara, A., Sekine, S., Yoshida, M., Yoshida, A., Taniguchi, H., Kushima, R., Tsuda, H. & Kanai, Y. (2013). *Histopathology* **62**, 657-661.

McGuire, M. M., Yatsenko, A., Hoffner, L., Jones, M., Surti, U. & Rajkovic, A. (2012). *PLoS One* **7**, e33251.

Osinovskaya, N. S., Malysheva, O. V., Shved, N. Y., Ivashchenko, T. E., Sultanov, I. Y., Efimova, O. A., Yarmolinskaya, M. I., Bezhenar, V. F. & Baranov, V. S. (2016). *Int J Gynecol Pathol* **35**, 509-515.

Perot, G., Croce, S., Ribeiro, A., Lagarde, P., Velasco, V., Neuville, A., Coindre, J. M., Stoeckle, E., Floquet, A., MacGrogan, G. & Chibon, F. (2012). *PLoS One* **7**, e40015.

Ravegnini, G., Marino-Enriquez, A., Slater, J., Eilers, G., Wang, Y., Zhu, M., Nucci, M. R., George, S., Angelini, S., Raut, C. P. & Fletcher, J. A. (2013). *Modern pathology : an official journal of the United States and Canadian Academy of Pathology, Inc* **26**, 743-749.

Rieker, R. J., Agaimy, A., Moskalev, E. A., Hebele, S., Hein, A., Mehlhorn, G., Beckmann, M. W., Hartmann, A. & Haller, F. (2013). *Pathology* **45**, 388-392.

Sadeghi, S., Khorrami, M., Amin-Beidokhti, M., Abbasi, M., Kamalian, Z., Irani, S., Omrani, M., Azmoodeh, O. & Mirfakhraie, R. (2015). *Tumour Biol*.

Schwetye, K. E., Pfeifer, J. D. & Duncavage, E. J. (2014). *Human pathology* **45**, 65-70.

Ye, J., Wang, H., Chen, Y. B., Yuan, D. L., Zhang, L. X. & Liu, L. (2015). *Oncol Lett* **9**, 2771-2774.
